# Therapeutic types and advantages of functionalized nanoparticles in inducing ferroptosis in cancer therapy

**DOI:** 10.1080/07853890.2024.2396568

**Published:** 2024-09-14

**Authors:** Ziying Wang, Miaomiao Zhao, Xiaotong Huang, Yuxin Wang, Wentong Li, Jianhong Qiao, Xiao Yang

**Affiliations:** a School of Nursing, Shandong Second Medical University, Weifang, Shandong, China; b Department of Pathology, Shandong Second Medical University, Weifang, Shandong, China; c School of Pharmacy, Binzhou Medical College, Yantai, Shandong, China; d Department of Outpatient, The First Affiliated Hospital of Shandong First Medical University & Shandong Provincial Qianfoshan Hospital, Jinan, Shandong, China

**Keywords:** Ferroptosis, nanoparticles, cancer, drug delivery system

## Abstract

**Background:**

The clinical efficacy of cancer treatment protocols remains unsatisfactory; however, the emergence of ferroptosis-driven therapy strategies has renewed hope for tumor treatment, owing to their remarkable tumor suppression effects. Biologically based small-molecule inducers are used in conventional method to induce ferroptosis. Nevertheless, some molecular drugs have limited solubility, poor ability to target cells, and fast metabolism, which hinder their ability to induce ferroptosis over a prolonged period. Fortunately, further investigations of ferroptosis and the development of nanotechnology have demonstrated that nanoparticles (NPs) are more efficient in inducing ferroptosis than drugs alone, which opens up new perspectives for cancer therapy.

**Objective:**

In order to organize a profile of recent advance in NPs for inducing ferroptosis in cancer therapy, and NPs were comprehensively classified in a new light.Materials and methods: We comprehensively searched the databases such as PubMed and Embase. The time limit for searching was from the establishment of the database to 2023.11. All literatures were related to “ferroptosis”, “nanoparticles”, “nanodelivery systems”, “tumors”, “cancer”.

**Results:**

We summarized and classified the available NPs from a new perspective. The NPs were classified into six categories based on their properties: (1) iron oxide NPs (2) iron - based conversion NPs (3) core-shell structure (4) organic framework (5) silica NPs (6) lipoprotein NPs. According to the therapeutic types of NPs, they can be divided into categories: (1) NPs induced ferroptosis-related immunotherapy (2) NPs loaded with drugs (3) targeted therapy of NPs (4) multidrug resistance therapy (5) gene therapy with NPs (6) energy conversion therapy.

**Conclusions:**

The insights gained from this review can provide ideas for the development of original NPs and nanodelivery systems, pave the way for related nanomaterials application in clinical cancer therapy, and advance the application and development of nanotechnology in the medical field.

## 1. Introduction

Despite significant advancements in scientific research to diagnose and treat malignant tumors during recent decades, it remains a significant global health problem, leading to severe morbidity and mortality [1,2]. Currently, surgery, radiotherapy, and adjuvant chemotherapy are the primary clinical therapeutic modalities for cancer [3]. However, these approaches are limited by a few crucial drawbacks such as low response rates, medication resistance, and adverse drug reactions. The efficacy of cancer therapy still lags behind the expectations of patients for a healthy and long life. Therefore, there is a pressing need for breakthroughs in cancer treatment [4].

Recent researches have unveiled that ferroptosis, a new subtype of non-apoptotic cell death, has significant potential for effective cancer treatment [5,6]. Lethal lipid peroxidation (LPO), which builds up as a result of iron-dependent metabolism, upsets cellular redox equilibrium and results in cell death [7,8]. Furthermore, ferroptosis offers various benefits, including the ability to induce metabolic reprogramming, enhance immunogenicity, and limit tumor spread by affecting REDOX homeostasis [9]. Various drugs, such as molecular inhibitors, gene therapies, and nanodrugs, have been developed to induce ferroptosis [10–14].

Nevertheless, it is challenging for molecular medicines to induce ferroptosis over an extended period, due to their poor solubility, limited capacity to target specific cells and rapid metabolism. To prevail these limitations, scientists have exploited techniques to trigger ferroptosis using nanomaterials [15]. NPs-induced ferroptosis has gained significant attention and is considered a promising strategy for cancer treatment [16]. Liposomes, micelles made of polymers (PMs), dendrimers in quantum dots (QDs), nanotubes made of carbon (CNTs), mesoporous NPs made of silica (MSNs), metallic/magnetic NPs and quantum dots (QDs) are among the nanomaterials/NPs being explored as potential cancer treatments [17,18]. These intriguing nanomaterials/NPs offer tailored medication delivery, regulated release, enhanced stability, and heightened penetration into tumor tissue [17]. They also enable photothermal therapies and multimodal imaging. Furthermore, nanomaterials/NPs have the capability to selectively target cancer cells by modifying and functionalizing their surfaces. This enhances the administration of drugs while minimizing their negative side effects. Although the production of NPs poses challenges, continued investigation and advancement show significant potential for utilizing nanotechnology in the treatment of cancers [18].

In this review, we used ‘ferroptosis’, ‘nanoparticles’, ‘nanodelivery systems’, ‘tumors’, ‘cancer’ as the search terms, and comprehensively searched the databases such as PubMed and Embase. The time limit for searching was from the establishment of the database to 2023.11. We read the titles and abstracts to exclude the irrelevant and repetitive literature on the topics, and read the full text to re-screen the literature to further exclude the irrelevant literature. Here, we adumbrate latest progress in the types of NPs that induce ferroptosis in various cancer treatments. In addition, we summarize and classify the available NPs from a new perspective. The NPs were classified into six categories based on their properties: (1) iron oxide NPs (2) iron - based conversion NPs (3) core-shell structure (4) organic framework (5) silica NPs (6) lipoprotein NPs. According to the therapeutic types of NPs, they can be divided into categories: (1) NPs induced ferroptosis-related immunotherapy (2) NPs loaded with drugs (3) targeted therapy of NPs (4) multidrug resistance therapy (5) gene therapy with NPs (6) energy conversion therapy. Meanwhile, this review presents mechanism of NPs-induced ferroptosis therapy, which will contribute to our growing understanding of ferroptosis. In addition, this review show that nanomaterials/NPs have great application potential in the biomedical field. NPs initiate immunotherapy by activating the innate immune system to identify and eliminate tumor cells, while causing minimal harm to healthy tissue [19]. Loading drugs into NPs significantly improves delivery efficiency and reduces adverse reactions [20]. By targeting tumor cells, NPs can accumulate in cancer cells in high concentration, improving the effective concentration of drugs within cancer cells and enhancing the anti-tumor effect [21]. Some NPs loading light or sound sensitizers can perform tumor imaging while performing photokinetic or acoustic therapy based on ferroptosis [22]. These emphasize versatility and efficiency of NPs, fostering advancement in biomedical research. In short, the utilization of nanomaterials/NPs in the treatment of cancer offers patients safer and more efficient therapeutic alternatives, facilitating the implementation of related nanomaterials in clinical cancer therapy. This development is anticipated to yield novel breakthroughs and advancement in the realm of cancer therapy.

### 1.1. Classification of NPs properties

Nanotechnology has attracted extensive concern in realm of tumor diagnosis and treatment, owing to the novel tools it provides to address issues with traditional treatment. There are numerous types of nanomaterials, including metals, carbon/silica-based materials, polymers, ceramics, and hybrids, whose shape, size, and properties could be uniquely customized for various applications [23]. Most commonly, these nanomaterials are used as carriers for medicines, monoclonal antibodies, proteins, and nucleic acids [24]. NPs are important components of nanomaterials. In recent years, NPs have garnered significant attention for inducing ferroptosis. However, owing to their diverse properties, there is a lack of a certain summary, so the properties of NPs are briefly classified.

#### 1.1.1. Iron oxide NPs

Iron oxide NPs (IONPs), which are applied as drug carriers for treatment of cancers and contrast agents for magnetic resonance imaging, have been licensed by the Food and Drug Administration (FDA) [25,26]. For instance, pH-sensitive and decomposable theranostic superparamagnetic iron oxide NPs (SPIONs) have been established for cancer imaging and administration [27]. In acidic TME, SPIONs broke down into smaller NPs and released iron ions. In addition, under X-ray irradiation, nanoclusters were activated by the TME, resulting in cell death through a synergistic ferroptosis/apoptosis pathway. The anti-cancer activity of innovative magnetic IONP (IONP-GA/PAA) functionalized with gallic acid (GA) and polyacrylic acid (PAA) was assessed [28], which accurately adjusted the physicochemical characteristics that induced intrinsic anticancer activity through ferroptotic reactions. Ferumoxytol, a clinical-grade iron oxide NP, has been approved as an iron supplement in individuals with renal failure in the United States [29,30]. The interaction between natural killer (NK) cells and cancer cells during ferroptosis mediated by ferumoxytol enhanced synergistic anti-cancer effect of ferroptosis for prostate cancer cells bound to NK cells, enhancing the cytotoxic of NK cells, implying that NK cell function was improved *via* ferroptosis by ferumoxytol [31].

#### 1.1.2. Iron - based conversion NPs

**Figure 1.**
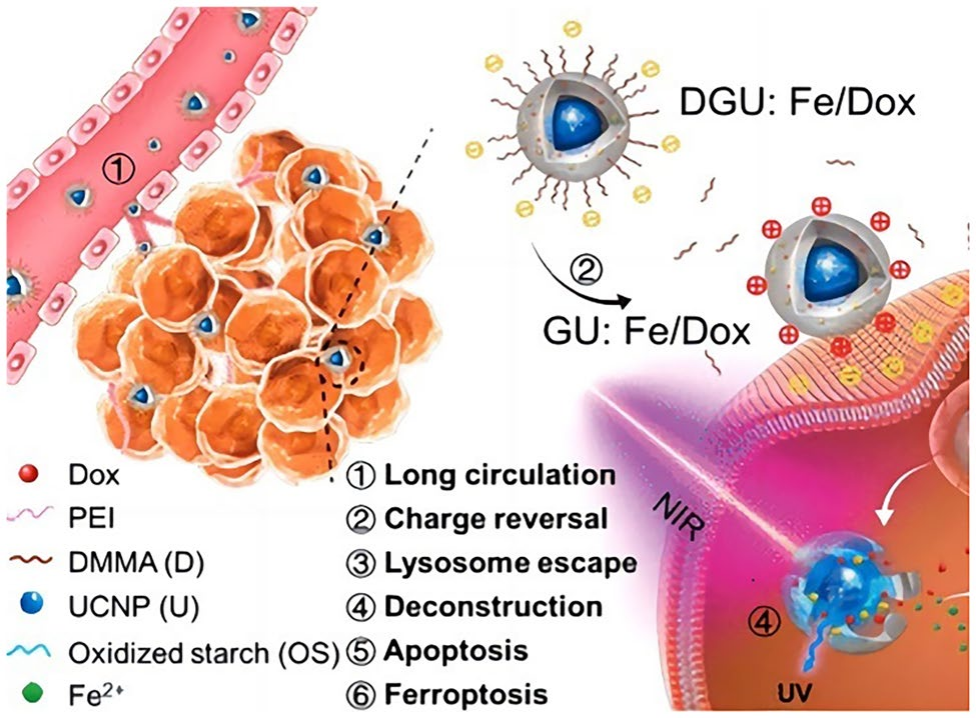
Schematic diagram Synthesis of polytransformed nalongan and related anticancer mechanism. (A) Structure of polytransformed nalongan. (B) Anticancer mechanism: under near-infrared radiation, nanolongan disintegrated, releasing Dox and Fe^2+^, thereby inducing cell apoptosis and ferroptosis. Reproduced with permission. Copyright 2019, American chemical society.

It has been suggested that ultraviolet/visible (UV/vis) light irradiation could accelerate the Fenton reaction and efficiently increase reactive oxygen species (ROS) generation. Nevertheless, serious damaging effects, poor tissue penetration, and fast attenuation restrict the application of UV light [32]. Long-wavelength near-infrared (NIR) light with properties of minor harm to living tissues and noticeably deeper tissue penetration could be transformed into shorter wavelength UV/vis light by UCNPs, which were developed to improve Fenton reaction and increase the effectiveness of ferroptosis. For instance, a nanolongan delivery system with a variety of on-demand conversion functions was designed, which contained UCNPs and doxorubicin (Dox) in a colloidal particle composed of Fe^3+^-cross-linked oxidized starch [33]. Surface charge conversion of nanolongan increased circulation time to take advantage of increased retention and permeability effects, enabling effective ingestion by cancer cells accompanied by lysosomal escape. Conversion of UCNPs from NIR to ultraviolet light overcame the problem of shallow penetration depth and made it possible to convert Fe^3+^ to Fe^2+^. Accordingly, this valence change could cause deconstruction of the nanolongan gel networks, resulting in quick release of Fe^2+^ and doxorubicin (Dox). In this instance, intracellular H_2_O_2_ and Fe^2+^ underwent Fenton reaction to generate powerful ROS, inducing ferroptosis; meanwhile, co-released Dox entered the nucleus and synergistically caused more severe cell death (Figure 1).

#### 1.1.3. Core-shell structure

Core/shell NPs are gaining increasing attention because of their position at the forefront of materials chemistry and various other fields including as electronics, biology, pharmaceuticals and catalysis. Owing to the various coatings of the shell material, the properties of the core particles (e.g. reduced reactivity or thermal stability) can also be changed, improving the overall stability and dispersion of the NPs [34]. A prospective nanoplatform of Fe_3_O_4_@PGL NPs with a core/shell structure and good prospects was used for ferroptosis complementing photodynamic therapy (PDT) [35]. Fe_3_O_4_@PGL NPs were demonstrated to have excellent biocompatibility, negligble dark toxicity, high photosensitizer (PS) loading effectiveness, and a comparatively small size. Fe_3_O_4_@PGL NPs stimulated significant ROS formation by the Fenton reaction. Additionally, the existence of macrophages enhanced the development of ROS, as confirmed by fluorescence imaging. In addition, under laser irradiation, PDT promoted oxidative stress and Fenton reaction in macrophages, increasing anticancer efficacy by generating more ROS. Experiments have indicated that cancer cells were efficiently eradicated when exposed to Fe_3_O_4_@PGL NPs and light irradiation. Additionally, tumor growth was entirely suppressed by boosting PDT. Biomimetic magnetic Fe_3_O_4_-SAS@PLT NPs were produced by combining sulfasalazine (SAS)-loaded mesoporous magnetic NPs and camouflaging of the platelet membrane [36]. SAS not only inhibited inflammatory cell migration and the IκB kinase path [37,38], but also repressed cysteine absorption to decrease tumor development and lead to ferroptosis [39]. Fe_3_O_4_ NPs also induced ferroptosis, which worked synergistically with SAS in time and space. The platelet membrane coating enabled Fe_3_O_4_-SAS@PLT immune evasion and tumor metastatic targeting. Fe_3_O_4_-SAS@PLT NPs triggered ferroptosis by inhibiting the XC^-^ (xCT) transporter pathway [36].

As an oxygen carrier, perfluorooctyl bromide was enclosed within MnOx to build PFOB@MnOx NPs core/shell NPs (PM-CS NPs), which displayed powerful anticancer activity by O_2_-mediated ROS overproduction, LPO buildup, glutathione (GSH) depletion, and glutathione peroxidase 4 (GPX4) inactivation [40]. Furthermore, the supply of oxygen inhibited intracellular hypoxia inducible factor-1 (HIF-1), which further reduced the storage of lipid droplets (LDs) and released free polyunsaturated fatty acids (PUFA) to encourage LPO. Following PM-CS NPs treatment, acyl-CoA synthetase long-chain family member 4 (ACSL4) also increased, which enhanced ferroptosis sensitivity.

#### 1.1.4. Organic framework

##### 1.1.4.1. Metal-organic frameworks (MOFs)

MOFs are a kind of crystalline materials that have been developed for catalysis, gas storage, and sensing [41]. Due to their high loading capacity and biodegradation, MOFs are ideal platforms for drug transport and cancer treatment [42–44].

###### 1.1.4.1.1. MIL-101(Fe) MOF

**Figure 2.**
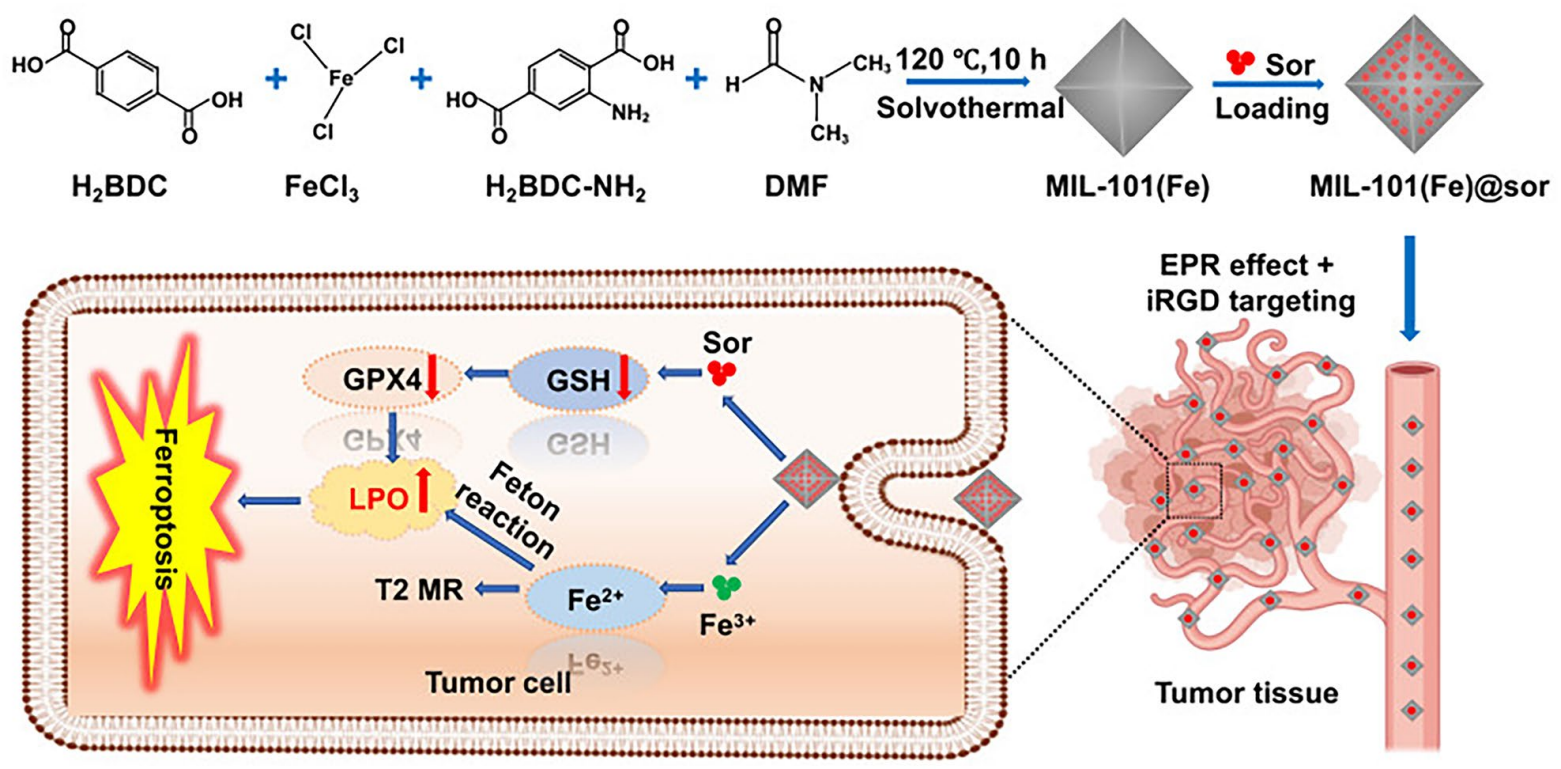
Synthesis And anticancer mechanism of MIL-101(Fe)@sor NPs. Reproduced with permission. Copyright international journal of nanomedicine 2021 16 1037-1050’ originally published by and used with permission from dove medical press ltd.

MIL-101(Fe) NPs exhibit a number of desirable properties, including the capacity for T2 magnetic resonance imaging, drug loading and biocompatibility. Sorafenib (SRF) was selected and loaded into MIL-101(Fe) NPs to build MIL-101(Fe)@sor [45]. MIL-101(Fe)@sor NPs greatly induced iron metabolism in HepG2 cells, increasing LPO and malondialdehyde (MDA), and reducing GSH and GPX4 (Figure 2). Similarly, MIL-101(Fe)@RSL3 was devised in which RSL3 (a ferroptosis inducer) was loaded with Iron-rich MIL-101 (Fe) NPs for targeting delivery and responding quickly to release [46]. In an acidic TME, progressive breakdown of MIL-101(Fe)@RSL3 released Fe^3+^ and RSL3, which aggravates ferroptotic cell death in cancer cells by causing iron overload and blocking GPX4.

###### 1.1.4.1.2. Zeolite imidazolate frameworks (zifs)

Zifs are among the most extensively researched nanocarriers, among which Zif-8 is a porous composite material consisting of Zn^2+^ and 2-methylimidazole [47–49]. Several benefits, including favorable biocompatibility, high porosity, and breakdown at low pH, have caused its widespread use as a drug carrier [50]. The weak coordination bonds of Zifs facilitate the sensitivity to acidic environment, and brittleness of these bonds make them appropriate for cancer diagnostic and therapeutic systems [51]. Zif-8 has grown into a versatile system in earlier research that plays a special and important role by displaying more functions in each layer [52,53].

**Figure 3.**
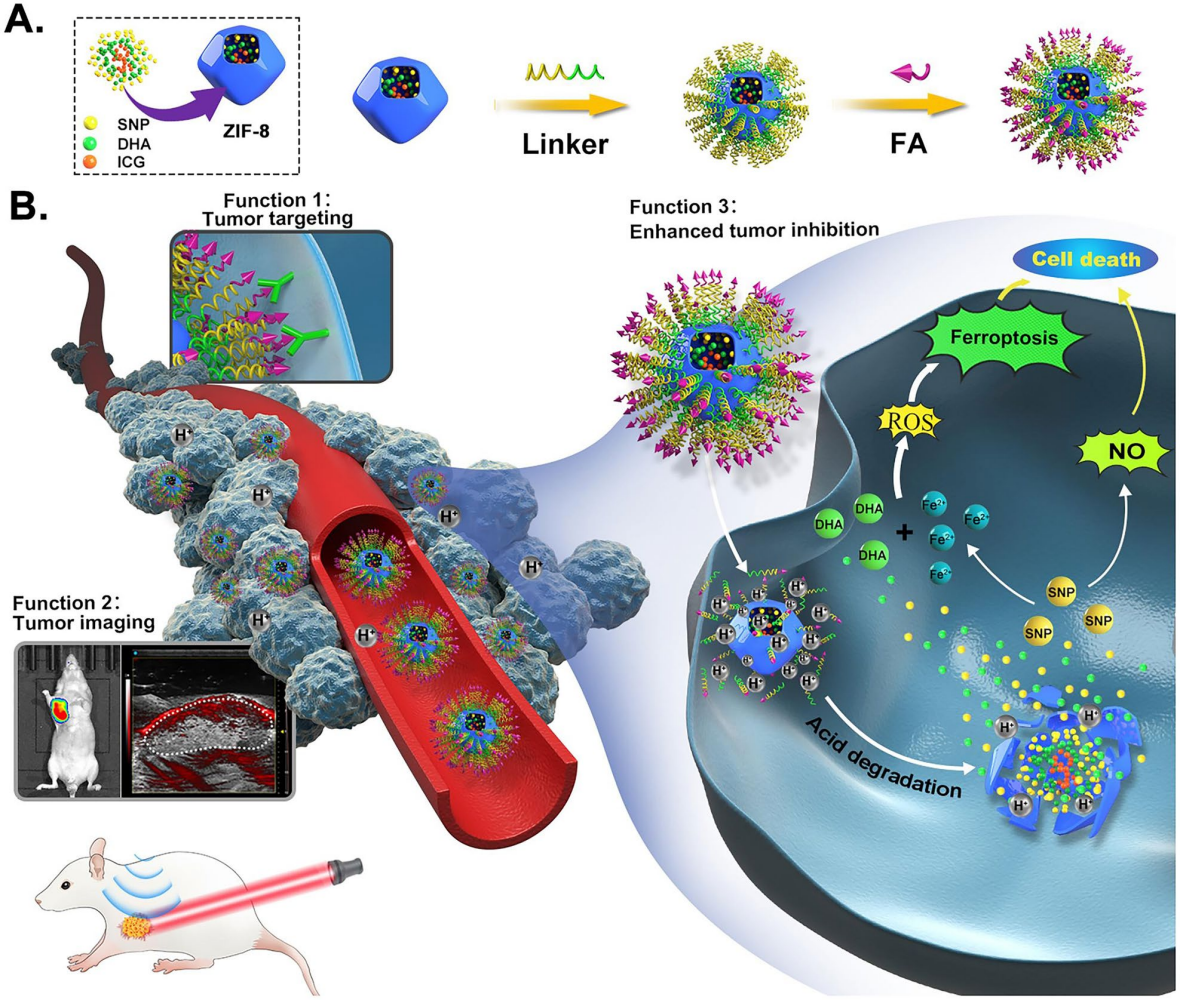
Synthesis And functional diagram of DHA/SNP/ICG@Zif-8-FA. (A) Synthesis diagram of nanoparticles. (B) Functions of nanoparticles: 1. Folate receptors (FA) promote cancer cell targeting by nanoparticles. 2. Nanoparticles are helpful for tumor diagnosis and localization. 3. Enhanced tumor ferroptosis. Reproduced with permission. Copyright 2021, elsevier.

DHA/SNP/ICG@Zif-8-FA containing dihydroartemisinin (DHA), folic acid (FA), sodium nitroprusside (SNP), indocyanine green (ICG), and Zif-8 was developed. Fluorescence and photoacoustic imaging devices can detect them and precisely pinpoint tumors when the NPs were internalized [54]. Additionally, following phagocytosis, pH-sensitive Zif-8 particles started to break down in lysosomes. SNP triggered Fe^2+^ and DHA simultaneous release as a donor of Fe^2+^/NO, while NO released by SNP inhibited tumors. GSH and GPX4 levels were also downregulated owing to synergistic effects. The combined effects of DHA and SNP caused explosive release and overgeneration of ROS to induce ferroptosis, which fatally harmed tumor cells (Figure 3). Similarly, GGF@ZIF-8@HA and Dox were synthesized by encapsulating glucose oxidase (GOx) and a mixture of gallic acid (GA), Fe(II)(GA/Fe) nanocomplexes (GGF), Dox in one pot into ZIF-8 NPs, and the surface was modified with HA [55]. Acidic microenvironment within endosomes/lysosomes caused the degradation and release of encapsulated cargo. Released GOx consumed glucose and produced H_2_O_2_, which reacted with GA/Fe to generate poisonous ·OH. Besides, released Dox induced chemotherapy-related tumor cell death.

##### 1.1.4.2. Covalent organic frameworks (COFs)

COFs are a kind of extended crystalline porous polymers and a type of organic carrier constructed by periodic organic units linked by dynamic covalent bonds with specific geometric shapes [56]. COFs may be prospective nanocarriers that integrate a variety of functional components and have highly customized and regular nanopores. Nanoscale COFs have been used for many aspects in the fields of medication delivery [57,58], phototherapy [59], radiation [60], and immunotherapy [61].

Studies have demonstrated that COFs offered the chance to controllably introduce metals into NPs to avoid the latent toxicity of metal-rich inorganic materials [62]. RSL3@COF-Fc was fabricated using ferrocene (Fc) and RSL3 based on COFs [63]. When tumor cells endocytosed NPs, progressively released RSL3 blocked GPX4 and Fc caused LPO by inducing ·OH generation through Fenton-like processes. In the end, NPs destroyed the membranes of plasma, lysosomes, and mitochondria and resulted in ferroptosis of tumor cells while being less hazardous to normal cells.

### 1.5. Silica NPs

Because of Silica NPs tiny size, distinctive surface features, stable physicochemical properties, and superior biocompatibility, they are widely used in cosmetics, food, tissue imaging, and medication delivery. Silica NPs were demonstrated to have ferroptosis-inducing activity, and ultrasmall (<10nm diameter) PEG-coated silica NPs modified by melanin-targeting peptides were created to trigger ferroptosis in starved cancer cells and xenograft tumors in mice [64]. In addition, Silica NPs caused liver histological damage and hepatocellular ferroptosis, characterized by the accumulation of free ferrous iron, aggravation of phospholipid hydroperoxides (PL-OOH), and mitochondrial membrane/cristae rupture in L-02 cells [65].

### 1.6. Lipoprotein NPs

Lipoprotein-like NPs (Flip-NPs) were created, which is the first identified high-density lipoprotein (HDL) receptor that sustains cholesterol homeostasis in cells. Flip-NPs reduce GPX4 expression and augment accumulation of LPO in lymphoma cells, which promotes ferroptosis [66]. Recently, LDL NPs were designed with docosahexaenoic acid and natural ω-3 PUFA. Reconstituted carriers preserved the characteristics of circulating plasma LDLs, such as their identification and absorption by LDL receptors. Delivering LDL-DHA NPs to tumor cells provoked a perfect storm where PUFAs settled in cellular environment with redox metabolism and deviant iron [67]. Subsequently, depletion of GSH and suppressed activity of GPX drove ferroptosis. Furthermore, the study assessed the anticancer properties of LDL NPs in a group of HepG2 mice with xenografts. The findings revealed that tumors in the group treated with LDL-DHA were 95% smaller compared to tumors in the other groups. These findings offered fresh understanding of the molecular pathways that regulated the cytotoxicity of LDL-DHA in tumors.

## 2. NPs induced ferroptosis-related immunotherapy

Cancer immunotherapy can induce a systemic and continued immune response to inhibit tumour metastasis and regression, which has revolutionized the clinical management of cancer in recent decades [68]. With emphasis on the promising clinical therapeutic efficacy of all kinds of immunotherapy, attention has also been directed towards the potential interplay between ferroptosis and the immune system [31]. Various aspects of ferroptosis-related immunity have been investigated.

### 2.1. Alteration of M1/M2 macrophage polarization based on ferroptosis

In addition to classical ATP, HMGB1, CRT, and other DAMP molecules, KrasG12D and 8-hydroxyguanosine are released by ferroptosis in tumor cells. These molecules influence the functions and phenotypes of innate immune cells such as macrophages in the TME. [69]. Macrophages can be affected by a variety of factors to polarize into M1 and M2 phenotypes based on the environment. M1 releases type I pro-inflammatory cytokines and exerts anti-tumor effects. In contrast, activated M2 contributed to tumor-promoting inflammation [70,71]. Studies have shown that polarization of tumor-associated macrophages from immunosuppressive M2 to anticancer M1 inhibits tumor growth [23]. It’s worth noting that there is an interaction between ferroptosis and the tumor microenvironment, which influences macrophage polarization.

**Figure 4.**
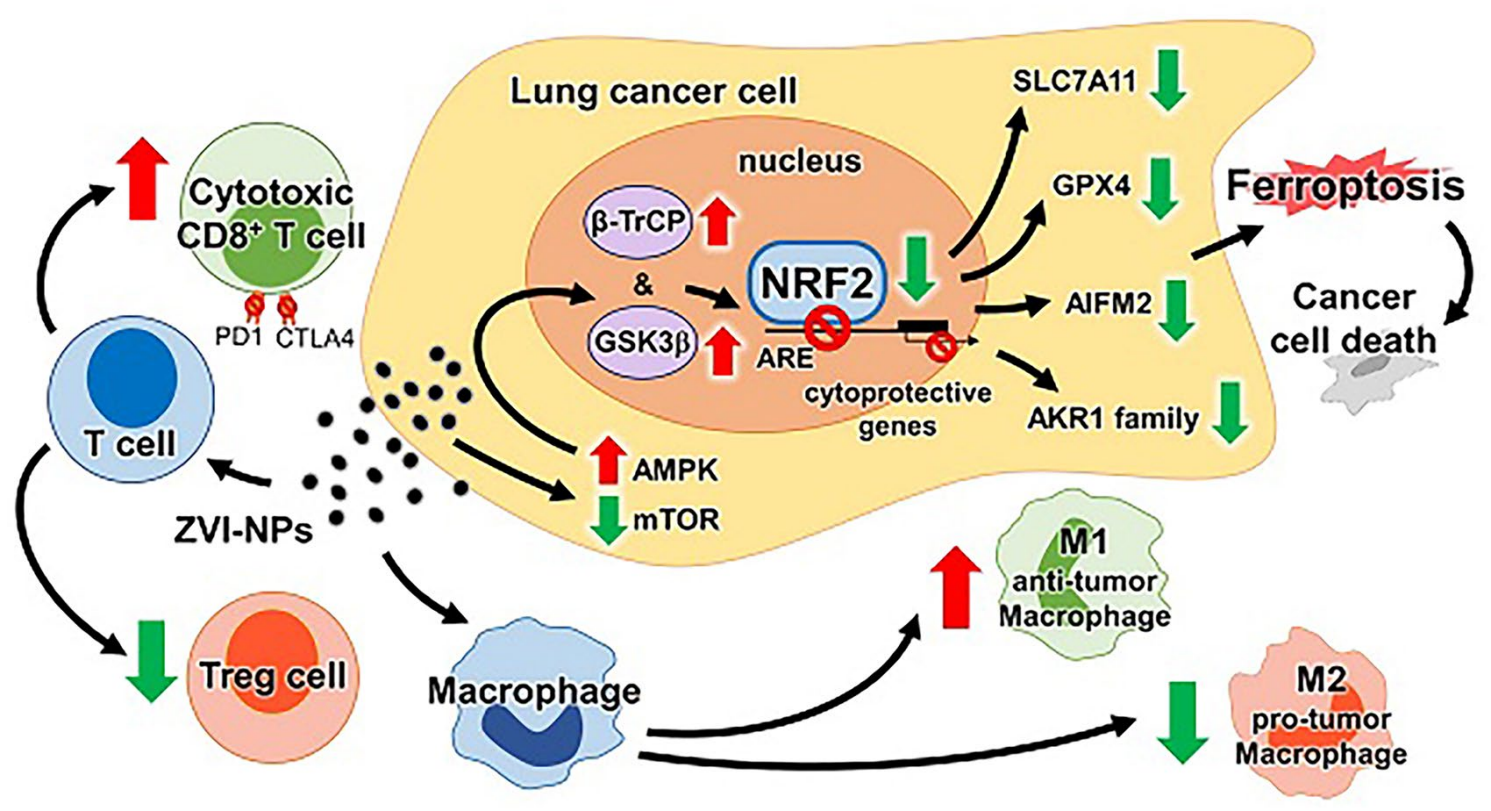
ZVI-NPs Nanoparticles can induce ferroptosis and trigger an immune response in combination with induced cell death. Reproduced with permission. Reproduced with permission. Copyright 2021, ivyspring international publisher.

All types of NPs have been studied for their potential to modulate tumor microenvironment. For instance, manganese dioxide NPs and magnetic NPs have been reported to reprogram macrophages, while manganese-based NPs have been found to enhance the expression of pro-phagocytic calreticulin, which facilitates the immune recognition of cancer cells [72]. Despite these promising results, the development of anticancer nanomedicine still faces challenges related to biocompatibility and large-scale production. To address these issues, zero-valent iron (ZVI) NPs have been developed that possess many advantages, such as generating ROS, promoting the Fenton reaction, and degrading organic contaminants for environmental cleanup [73]. It was recently reported that superior ROS-inducing properties resulted in mitochondria malfunction, intracellular oxidative stress and LPO, making ZVI NPs become potential bactericides and anticancer agents [74–76]. ZVI-NPs activated anti-tumor immune responses by modulating the polarization of macrophages from M2 to M1 phenotype, abating population of regulatory T cells, and decreasing expression of PD-1 and CTLA4 in CD8^+^ T cells to boost cytotoxicity against cancer cells. In addition, ZVI-NPs selectively triggered ferroptosis by efficiently delivering large amounts of iron to cancer cells by enhancing the degradation of Nrf2 [72] (Figure 4).

The platelet membrane coating provided Fe_3_O_4_-SAS@PLT immune evasion and tumor metastatic targeting. The NPs induced a resultful immunoreaction and increased PD-1 blockade therapeutic value *in vivo*. Fe_3_O_4_-SAS@PLT triggered ferroptosis *via* inhibiting the Xc − pathway [77]. Proteomic analysis revealed that Fe_3_O_4_-SAS@PLT-induced ferroptosis repolarized macrophages from M2 to M1 [36]. These indicated that Fe_3_O_4_-SAS@PLT reinforced systemic anti-tumor immunity and provided a novel way for clinical application of synergistic immunotherapy in tumors.

### 2.2. Increased T cell infiltration based on ferroptosis

ZnP@DHA/pyro-Fe core-shell NPs were designed for the co-delivery of Pyro-Fe and Chol-DHA to induce ferroptosis in an immunogenic manner to enhance anticancer efficacy [78]. DHA is one of the few immune cell death inducers, exhibiting cytotoxicity in various cancer cells and inducing ferroptosis. Free radicals produced by DHA oxidize lipids destroyed membrane proteins, thereby inducing cancer cell death. Enhancing the delivery of Chol-DHA and Pyro-Fe to tumors induced the accumulation of ROS, which inhibited tumor growth [78]. Furthermore, the combination of pyro-Fe and DHA enhanced DHA immunostimulation, which was proven by higher HMGB-1 release and increased CRT exposure. United treatment also increased intratumoral CD8^+^ T cell infiltration and enhanced anti-PD-L1 immune checkpoint blocking to induce cell death [78]. ZnP@DHA/Pyro-Fe NPs were administered into the CT26 colorectal tumor model, resulting in significant reduction of 65.1% in tumor weight and tumor growth inhibition index of 57.9%. All of these findings indicated that stimulating ferroptosis in an immunogenic manner can greatly enhance the effectiveness of anti-cancer treatment.

## 3. NPs loaded with drugs

### 3.1. Loaded with chemical drugs

#### 3.1.1. Loaded with chemotherapeutic drug

##### 3.1.1.1. Loaded with dox

Dox is an anti-tumor chemotherapeutic agent, and its cytotoxicity can produce negative effects. Clinical and preclinical studies have explored many types of formulations to reduce side effects, including encapsulation by nanomaterials, such as liposomes, proteins, and PLGA NPs [79–82].

**Figure 5.**
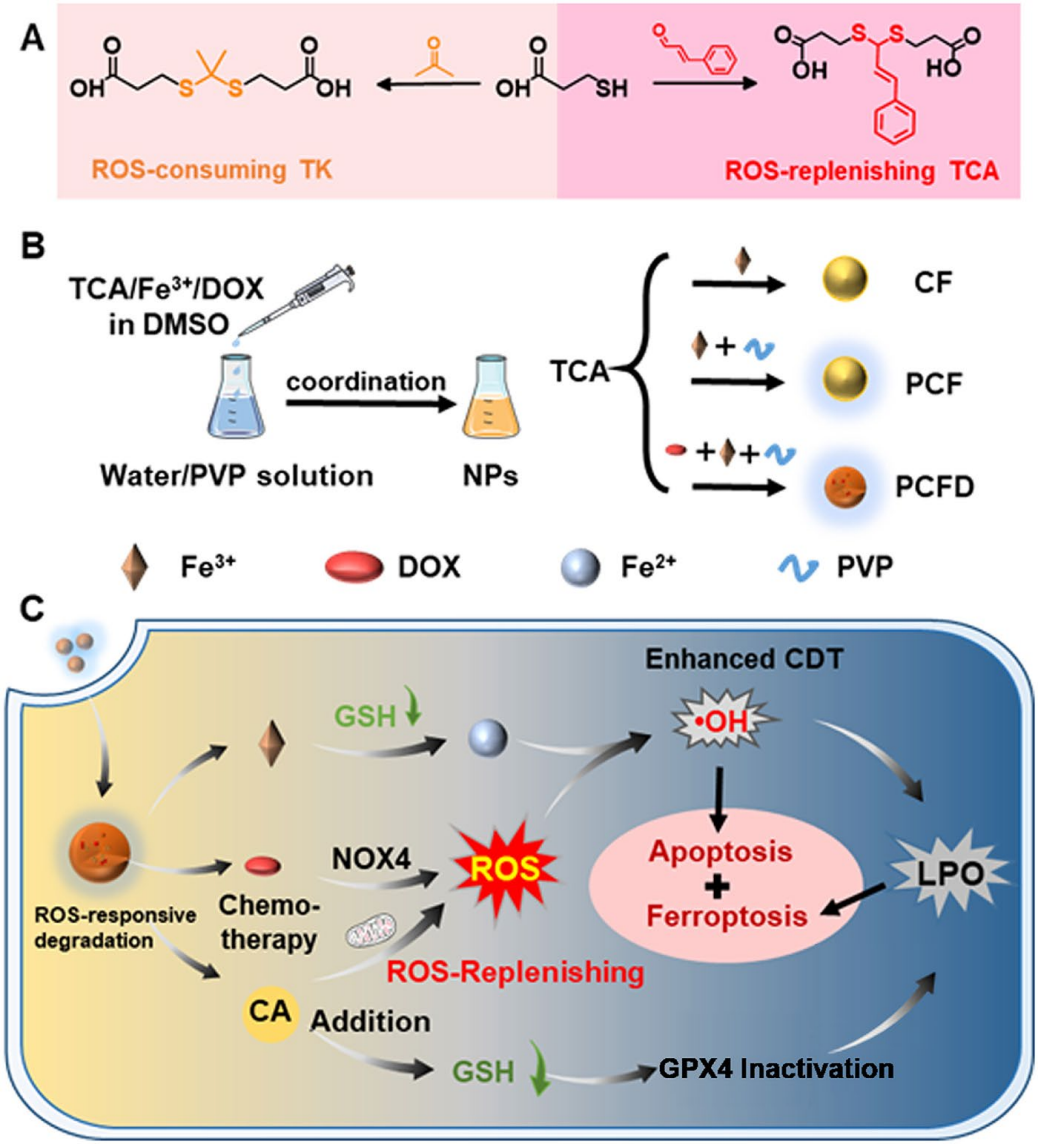
Synthesis Of PCFD and the mechanism of inducing cancer cell death. (A) Synthesis of TCA and TK. (B) Schematic diagram of the process used to prepare PCFD NPs. (C) Schematic diagram of the process by which PCFD induces cancer cell death *via* ferroptosis and apoptosis. Reproduced with permission. Copyright 2022, elsevier.

ROS-upregulating and GSH-consuming iron-based nanosystems have shown enormous potential in enhancing ferroptosis by producing abundant ·OH and destroying the redox homeostasis of cancer cells [83,84]. Therefore, an ROS-sensitive ligand doped with cinnamaldehyde (CA) was designed, and an iron-coordinated polymer NP loaded with Dox (PCFD) was constructed to disrupt the intracellular redox equilibrium and realize combinational chemo-chemodynamic cancer treatment [83]. Dox activated NADPH oxidase (NOX) in the mitochondria to produce ROS [85–88]. Upon internalization by cancer cells, PCFD released Dox, CA, and Fe^3+^ to augment ROS and consume GSH, causing persistent release of Dox, a large amount of ·OH generation, causing apoptosis and ferroptosis. The results of *in vivo* studies demonstrated a substantial tumor inhibition effect (59.6%) in the PCFD group. Additionally, histological examination using HE staining revealed a larger proportion of tumor necrosis and apoptosis in the PCFD group [89] (Figure 5).

##### 3.1.1.2. Loaded with cisplatin (Pt)

Pt causes cell cycle arrest and inter-and intra-strand DNA cross-linking by forming adducts with purine nucleobases [90]. Recent research has shown that Pt-loaded NPs have a greater potential to treat cancer by inducing ferroptosis on the basis of fully utilizing Pt drugs.

A Pt prodrug-loaded manganese-deposited iron oxide nanoplatform (Pt-FMO) was designed to induce cascade events that produced ROS and boosted the ferroptotic effect [91]. After the NPs were internalized, Pt-FMO generated the release of Mn, Pt(IV) prodrug, and Fe ions in response to the TME. In addition, Pt (IV) prodrugs were activated by endogenous GSH to produce Pt. While Mn^2+^ enhanced Fe^2+^/Fe^3+^ triggering ferroptosis, Pt can increase cell H_2_O_2_ levels and significantly enhance ferroptosis through intracellular Fenton reaction.

##### 3.1.1.3. Loaded with SRF

SRF, a small chemical kinase inhibitor, suppresses the growth of tumor cell and neovascularization by inhibiting the RAF/MEK/ERK signal path [92]. Additionally, SRF triggers ferroptotic death in cancer cells by inhibiting xCT, which causes ROS buildup [93]. Owing to its poor retention and short half-life, SRF alone cannot trigger tumor ferroptosis. Thus, loading SRF into NPs can enhance its therapeutic effects while retaining its benefits.

Polyvinylpyrrolidone-stabilized SRF-loaded copper peroxide (CuO_2_-PVP-SRF) NPs were produced for cancer chemodynamic treatment [94]. NPs have some advantages such as good intracellular degradation, pH-independent self-supplying Fenton-like ions, and GSH consumption capacity. Under acidic conditions, pH-sensitive CuO_2_ NPs progressively broke down into Cu^2+^ and H_2_O_2_, creating an augmented Fenton-like reaction. The released SRF stimulated the buildup of ROS by suppressing the function of system xc−, thereby initiating ferroptosis. Furthermore, the study revealed that the NPs induced a mortality rate of cancer cells at 45.47%, indicating promising potential for tumor therapy. Similarly, SRF was incorporated into mesoporous silica NPs (MMSNs) to generate MMSNs@SO NPs, which inhibited HepG2 tumor cells by depleting GSH, inhibiting intracellular GSH synthesis, and causing cancer cell ferroptosis [95]. In addition, SRF@FeShik-GOx-cRGD supramolecular nanomedicines (SNs) were designed using FeShik SNs as carriers to deliver GOx and sorafenib to achieve triple ferroptosis amplification [96]. FeShik released Fe^2+^ and depleted GSH, whereas GOx facilitated formation of ·OH *via* Fenton reaction by providing an acidic environment and a substantial quantity of H_2_O_2,_ which inhibited GSH biosynthesis by suppressing xCT and deactivating GPX4 [96]. Furthermore, SRF hindered the generation of GSH by blocking system Xc−, which also disabled the enzymatic function of GPX4. *In vivo*, the tumor inhibition rate of SRF@FeShik-GOx-cRGD SNs was 81.5%. The HE stained tumor sections exhibited the highest degree of tissue necrosis in comparison to the control groups. In short, the efficacy of NPs against 4T1 cells was enhanced in collaboration with RF-mediated ferroptosis and shikonin-mediated necrosis.

Magnetic NPs possess good biocompatibility and short blood circulation period. Consequently, the zwitterionic polymer poly (2-methacryloyloxyethyl phosphorylcholine) (PMPC) was changed by precipitation polymerization to form composite magnetic NPs (MNP) (MNP@PMPC). Then SRF were loaded into MNP@PMPC to build MNP@PMPC-SRF [97]. MNP offered a substantial quantity of iron to generate LPO, causing ferroptotic death of cancer cells. In addition, the disulfide cross-linked polymerization coat was degraded, and the released SRF down-regulated GPX4 expression in cancer cells, further promoting ferroptosis. Anti-tumor rate of MNP@PMPC-SRF group was the highest (84.1%) after 14 days, which could offer a superior alternative for enhancing the effectiveness of medicines that triggered ferroptosis.

##### 3.1.1.4. Loaded with salinomycin

Salinomycin is an effective anticancer drug that selectively kills stem cells by the generation of ROS to induce ferroptosis. To minimize toxicity and achieve smooth and continuous delivery of Salinomycin, a new biodegradable polymeric NPs (HSB-1216) was developed. HSB-1216 dramatically reduced acute myeloid leukemia cell clonogenic survival and killed venetoclax-resistant AML cells [98].

#### 3.1.2. Loaded with antibiotic

Sulfonamides (SAS) inhibited development of diverse tumor cells through suppressing xCT and inactivating GPX4 to trigger ferroptosis in preclinical models [99,100]. Fe(III)PP@SAS NPs were prepared by co-loading ferric ions and SAS onto PEGylated PDA *via* metal coordination and π-π stacking interactions [101]. The nanoplatform showed synergistic effects by triggering ferroptosis in tumor cells. In the endosome, ferrous ions subsequently induced Fenton reaction to produce ·OH radicals and fought against tumors. Released SAS inhibited xCT/GPX4, destroying the ‘shield’ that prevented tumor cells from ferroptosis. That showed potential for cancer treatment.

#### 3.1.3. Loaded with autophagy inducer

There has been growing concerns about combining different cell death pathways to treat cancer, and the combination of ferroptosis and autophagy has shown great potential [102,103]. A novel nanocarrier system that delivering an autophagy inducer (trehalose) and manganese oxide-integrated mesoporous silica (TreMMM) was developed for highly effective tumor treatment through autophagy-enhanced ferroptosis [104]. TreMMM triggered ferroptosis by inhibiting GPX4, which was achieved through the high GSH consumption of MnOx NPs. Additionally, trehalose released from TreMMM NPs induced autophagy to accumulate LPO, causing NCOA4-mediated degradation of ferritin, promoting ferroptosis. Similarly, Fe^3+^@erastin@rapamycin micelles (REFSM) were designed. REFSM dissociated in acidic conditions of the lysosome and enzyme system, allowing the release of Fe^3+^ and erastin to stimulate ferroptosis, which was reinforced by released rapamycin *via* irritating ferritinophagy and lipophagy, and inhibiting HIF-1α [105]. Furthermore, the study also assessed the anti-cancer efficacy of REFSM-treated 4T1 cells *in vitro*, confirming a significant reduction in cell survival with only 20% viability compared to the control groups. The tumor inhibition rate of REFSM was 64% *in vivo* experiments. Furthermore, therapy with REFSM has been found that had a more rapid and satisfied anticancer effect compared to the control groups. Simultaneously, histological examination using H&E staining revealed that REFSM treatment resulted in significant impairment to the tumor tissue.

#### 3.1.4. Loaded with GPX4 inhibitor

The majority of ferroptosis inducers enhance the buildup of LPO within cells by reducing the levels of GPX4. GPX4 inhibitor FIN56 was combined with arachidonic acid (AA) to form stable NPs. These NPs were then modified with PEG polymers to create LPO nanoamplifiers (FAS NPs). The purpose of this modification was to enable long-term circulation in the bloodstream and target release of the medicine specifically in tumours. FIN56 enhanced the buildup of LPO *via* blocking the activity of GPX4. Simultaneously, AA underwent oxidation, resulting in the production of abundant ROS, which synergistically worked with FIN56 to cause tumour ferroptosis [106].

### 3.2. Loaded with light therapy drugs

Chlorin e6 (Ce6) is considered a prospective photosensitizer, which is derived from live Chlorella ellipsoidalis and has powerful absorption in the red spectrum [107]. Ferritin-hijacking NPs (Ce6-PEG-HKN_15_) were prepared by coupling ferritin and the photosensitizer Ce6 to induce endogenous ferroptosis [108]. After internalization, the Ce6-PEG-HKN_15_ NPs selectively accumulated around ferritin. Under laser light, Ce6 that was activated produced significant quantities of ROS, which facilitated the degradation of iron storage proteins and activation of endogenous ferroptosis. The results of *in vitro* anti-cancer research showed a considerable increase in cell death proportion, reaching 65.96%. Additionally, *in vivo* experiments revealed that mice with 4t1 tumour growth, treated with Ce6-PEG-HKN15 NPs, exhibited the greatest survival rate. In addition, Fe_3_O_4_-PLGA-Ce6 NPs were designed using the photosensitizers Ce6 and Fe_3_O_4_. The Fe_3_O_4_-PLGA-Ce6 NPs dissociated in an acidic TME, releasing Ce6, and ferrous ions [109]. Under laser irradiation, the released Ce6 promoted ROS production and accumulation, promoting ferroptosis of 4T1 cells *via* photodynamic therapy. The study also investigated the anticancer properties, in live animal studies, it was shown that the Fe3O4-PLGA-Ce6 NPs + laser group had a tumour inhibition rate of 92.4%.

Protoporphyrin IX (PpIX) is generated in mitochondria [110], which has a powerful absorption capacity in the 380 to 650 nm wavelength range, and can be used for diverse applications, including photosensitizers for photodynamic therapy (PDT) [111]. PpIX-PSilQ NPs were designed to probe PDT cell death mechanism *in vitro*, with a particular focus on ferroptosis. The PDT action of PpIX-PSilQ NPs decreased the activity of GPX, which resulted in a decrease in the ability to scavenge peroxyl radicals and increase LPO levels in the treated cells, promoting the occurrence of ferroptosis [112]. This provided relevant results for the development of promising light-activated NPs.

### 3.3. Loaded acoustic therapy drugs

Exogenous ROS created by nanosonosensitizers during sonodynamic therapy (SDT) leads to significant ferroptosis, HSA-Ce6-IrO_2_ (HCIr) nanoclusters were designed which loaded sonosensitizer, Ce6 [113]. HCIr nanoclusters generated a large amount of ^1^O_2_ upon ultrasound (US) stimulation as nanosonosensitizers, which in turn increased intracellular LPO. Meanwhile, to prevent ROS scavenging, IrO_2_ depleted GSH through the self-cyclic fluctuation of Ir (III) and Ir (IV), which increased LPO levels by inactivating GPX4 to induce ferroptosis. Besides, experiments showed that the tumor inhibition rate of HCIr could reach 79.4%. In short, HCIr had capability to greatly enhance ferroptosis triggered by sonodynamics, resulting in powerful anti-tumor activity *in vivo*.

## 4. Targeted therapy of NPs

### 4.1. Cell membrane modification

Recently, biomembranes have been developed to disguise NPs, such as the outer membranes of cells and organelles. The membranes derived from erythrocytes, white blood cells, and platelets to modify nanodrug-carrying systems can not only evade identification of the immune system but also extend the period of circulation of drugs in the body [114,115]. Cancer cell membranes that inherit homologous targeting and antigenic reservoir from donor cells have been used for cancer-targeted treatment and cellular immunotherapy [116].

**Figure 6.**
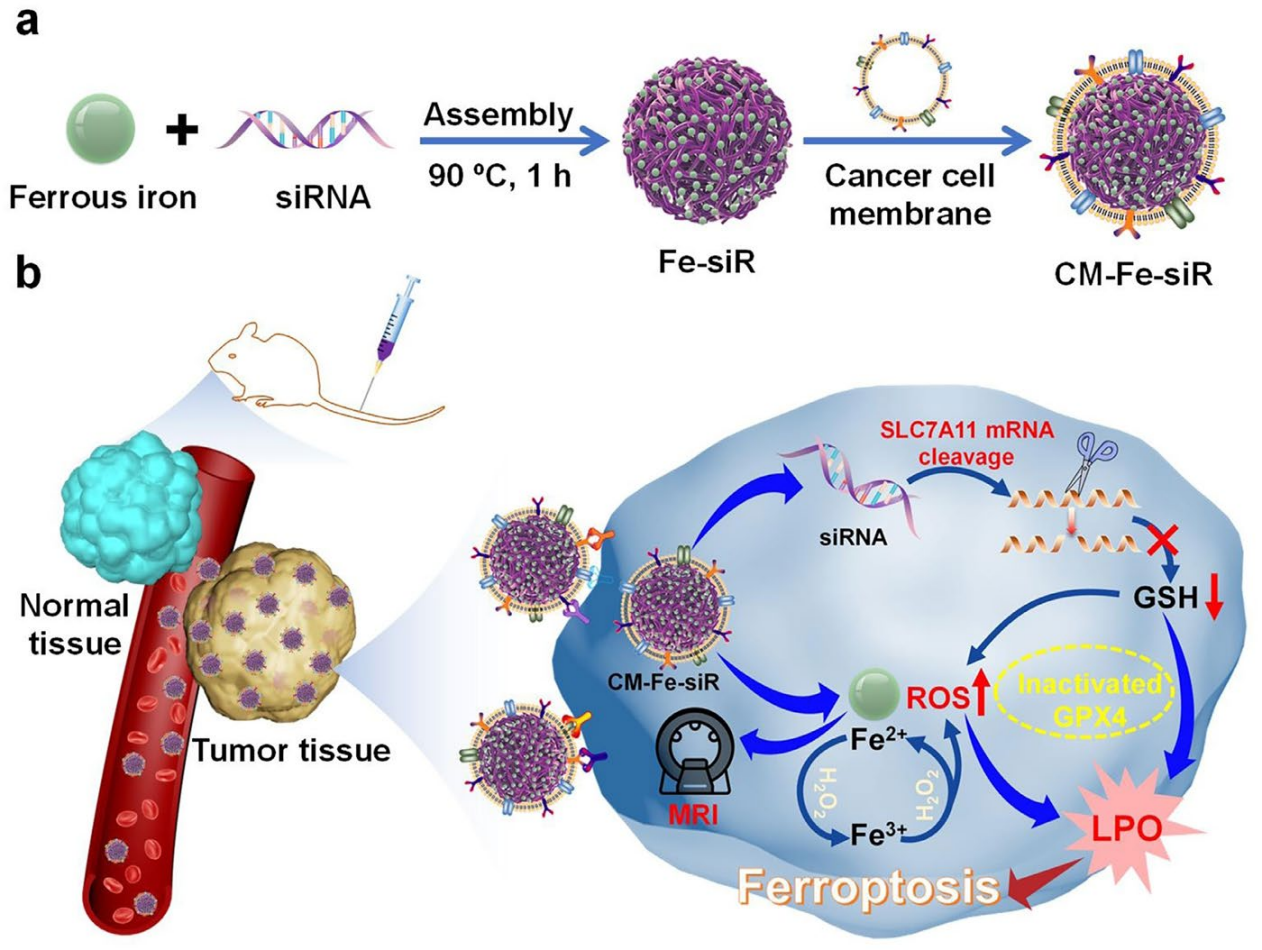
Synthesis And anticancer mechanism of CM-Fe-siR with targeted function (A) Synthesis process diagram of CM-Fe-siR. (B) The mechanism of ferroptosis induced by CM-Fe-siR. Reproduced with permission. Copyright 2022, elsevier.

Homologous tumor cell membrane-camouflaged iron-small interfering RNA nanohybridization (CM-Fe-siR) was developed for tumor-targeting, monitoring, and therapeutic functions [117]. The NPs were cleaved after endocytosis, releasing siRNA and ferrous iron, and siRNA targeting SLC7A11 blocked the uptake of cystine, which resulted in the reduction of GSH synthesis, and co-promoted LPO accumulation with iron-induced ROS production. Results from *in vitro* investigations demonstrated that the application of CM-Fe-siR caused a notable reduction in the average percentage of viable cells (58.2%). The therapeutic benefits of CM-Fe-siR nanohybrids were evaluated *in vivo* using CAL-27 tumour xenotransplantation mice models. The tumour weight of mice treated with Fe-siR, CM-FeSCR, and CM-Fe-siR was reduced by 30.3%, 15.2%, and 80.2%, respectively. To summarise, CM-Fe-siR exhibited pronounced cytotoxicity and considerable anti-tumor efficacy. DSA and SRF were used to chelate with Fe^2+^ to form DSe-SRF-Fe^2+^ nanodrug, coating with cancer cell membrane to build CM-DSe-SRF-Fe^2+^ to increase tumor targeting capability and enhance stability *in vivo*. In an acidic tumor microenvironment, Fe^2+^ release accelerated the intracellular Fenton reaction to promote ferroptosis. Simultaneously, the NPs resolved and liberated SRF, amplifying the synergistic impact. Furthermore, *in vivo* tests demonstrated a substantial tumour inhibition effect in the DSe-SRF-Fe^2+^ group (84%) [118] (Figure 6).

### 4.2. Folic acid (FA)

The FA receptor is overexpressed in most tumor cells, but is limited to healthy cells [21]. Therefore, FA, as an important cofactor in the synthesis of purines and pyrimidines and other cellular methylation reactions, is a suitable molecule for active targeting in cancer therapy. FA has been used for targeting delivery of drug-loaded NPs. FA-modified liposomes (FA-LP) were produced for selective delivery of MT1DP and erastin (E/M@FA-LPs) to improve drug/gene combination bioavailability and efficiency [119]. E/M@FA-LPs enhanced erastin-induced ferroptosis with depletion of cellular GSH and elevated ROS.

### 4.3. pH response

**Figure 7.**
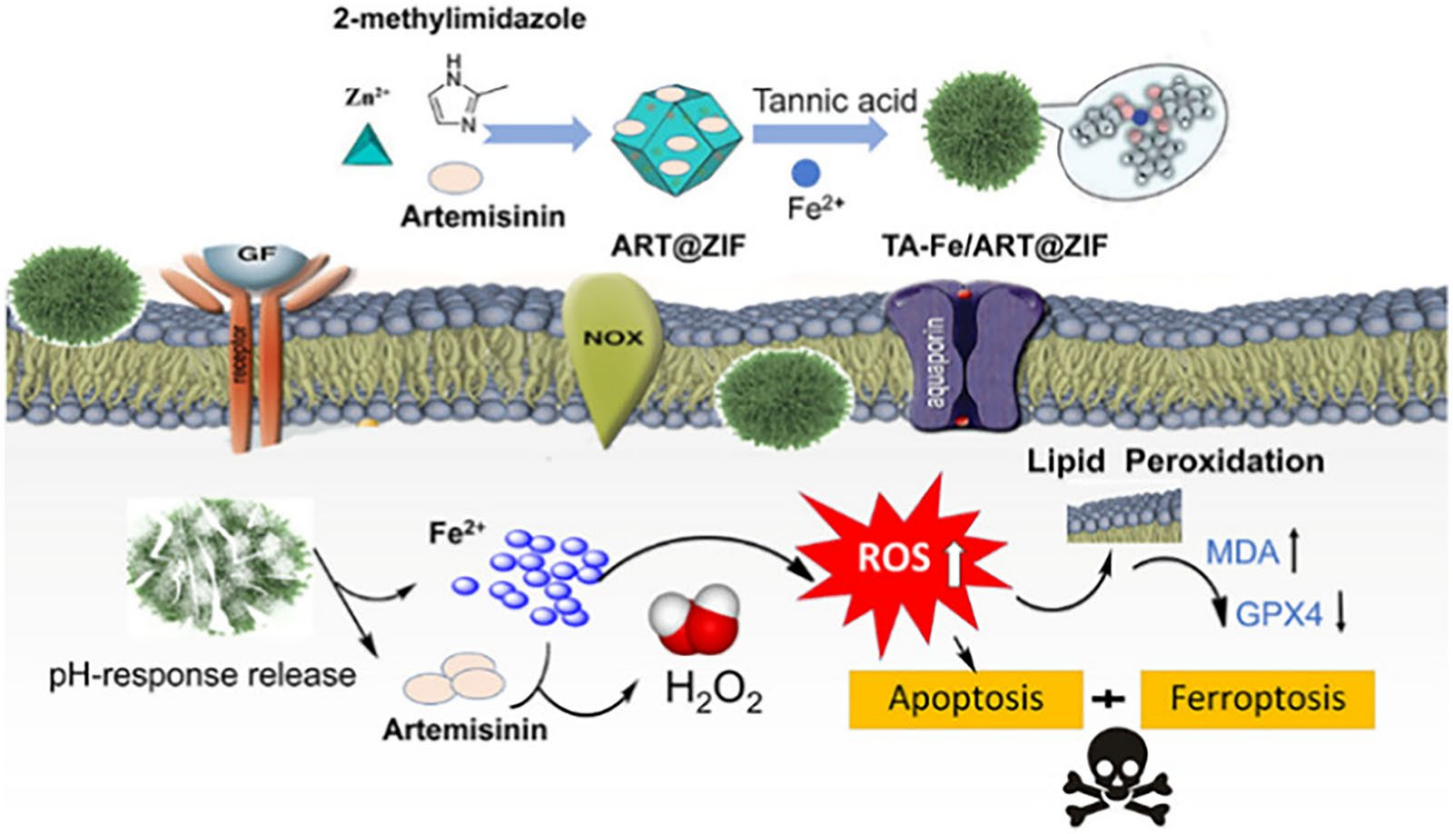
Schematic diagram of the preparation process of TA-Fe/ART@ZIF NPs and the mechanism of synergistic induction of tumor cell apoptosis/ferroptosis. Reproduced with permission. Copyright from Li, et al. 2021, springer nature.

Research has demonstrated that tumour tissues generate lactic acid and H^+^ in the surrounding microenvironment as a result of increased glycolysis caused by low oxygen levels. This leads to a slightly acidic pH in both the intra- and extracellular matrix of tumour tissues compared to normal tissues. The presence of this pH gradient enables the nanodelivery system to specifically target tumour cells. Additionally, pH-sensitive nanocarriers remain stable in normal tissues, but they can release drugs specifically in acidic conditions. This targeted drug release boosts the effectiveness of the drugs at the tumour site. Taking advantage of the properties of the PH response, a ferrous ion-tannic acid coordination-cloaked Zif-8 nanosystem encapsulating artemisinin (ART) (TA-Fe/ART@ZIF) was developed to regulate ferroptosis in cancer cells [120]. Biocompatibility and pH-responsive release make ZIF-8 become a promising nanocarrier for ART. After being absorbed by the cells, the nanosystem can be broken down in a slightly acidic microenvironment, leading to the release of Fe(II) and ART. This mechanism resulted in a substantial rise in intracellular ROS along with a decrease in GSH and GPX4 levels, which promoted ferroptosis and cell apoptosis. Experiment showed that the activity inhibition rate of TA-Fe/ART@ZIF NPs on MDA-MB-231 cells was 53.9%, which had a high tumor inhibition rate (Figure 7).

### 4.4. Magnetic targeting

Animal studies have shown that magnetic tumor targeting by iron oxide NPs (IONP) is possible [121]. Therefore, a multifunctional nanomagnetic vanadium (V)-iron-oxide (ION) NP (VIO) was synthesized, which could greatly enhance the accumulation of NPs in tumors, and superparamagnetic NPs could be externally directed to a certain position *via* a magnet. Mechanistic studies have confirmed that VIO-targeted tumors caused apoptosis and ferroptosis by increasing ROS levels [122].

## 5. Multidrug resistance therapy

### 5.1. Regulation of P-glycoprotein (P-gp) gene expression

The most frequent mechanism of MDR is super expression of P-gp, an ATP-dependent efflux pump with wide substrate optionality [123,124]. Based on this, Dox-Fe(VI)@HMS-HE-PEG (DFHHP) NPs were created by combining Dox with Fe(VI), followed by the addition of n-pentadiane, and finally modified with a polyethylene glycol (PEG) chain [125]. Under mild high temperature induced by US, released Fe(VI) reacted with water and overexpressed hydrogen peroxide in tumor cells to reoxidize independent of TME, significantly alleviating hypoxic TME. Reoxygenation suppressed P-gp and HIF-1 expression in tumor cells, sensitizing Dox. In addition, intracellular Fenton reaction were initiated by exogenous iron metabolism, resulting in overproduction of ROS and iron-dependent ferroptosis in cancer cells.

### 5.2. Reduce drug efflux

A Den-DOX-tannic acid-Fe^3+^ (DDTF) nanocarrier was created by combining a doxorubicin (DOX)-loaded dendrimer (Den) with a metal-phenolic network generated by Fe^3+^ and tannic acid. This nanocomplex combined a hybrid apoptosis/ferroptosis route to inhibit MDR in cancer cells [126]. The higher ROS levels induced by Dox made malignant cells more susceptible to ferroptosis. In addition, DDTF efficiently transported Dox to cancer cells by escaping multidrug efflux pumps *via* iron oxidation induced by Fenton reaction. Subsequently, superfluous ROS eventually killed the resistant cancer cells. This work utilised U14 and MCF-7/ADR Dutch nude mice to investigate tumour inhibitory capacity *in vivo*. DDTF had the highest tumour growth inhibition rate (91.7%) and apoptosis/necrosis rate (52.9%) when compared to the control groups. DDTF demonstrated superior efficacy *in vivo*. This could be attributed to its exceptional capacity to trigger apoptosis/ferroptosis.

### 5.3. Loaded with drug-resistant inhibitors

It is possible to transmit drug resistance inhibitors to tumor cells by encapsulating them in nano-sized carriers to reduce detrimental effects on healthy tissues and cells. Celecoxib (CXB) increases cancer cell sensitivity by suppressing P-gp expression. Buthionine ­sulfoximine (BSO) is a powerful and selective inhibitor of GSH synthesis that selectively interacts with γ-glutamylcysteine synthetase and improves the cytotoxic effects of several medications [127,128]. BSO/CXB@BNP hybrid NPs were designed to deliver CXO and BSO to reverse multidrug resistance. BSO/CXB@BNP showed higher efficiency in downregulating P-gp, diminishing GSH, and reversing drug resistance [129].

## 6. Gene therapy with NPs

### 6.1. Promote gene expression

**Figure 8.**
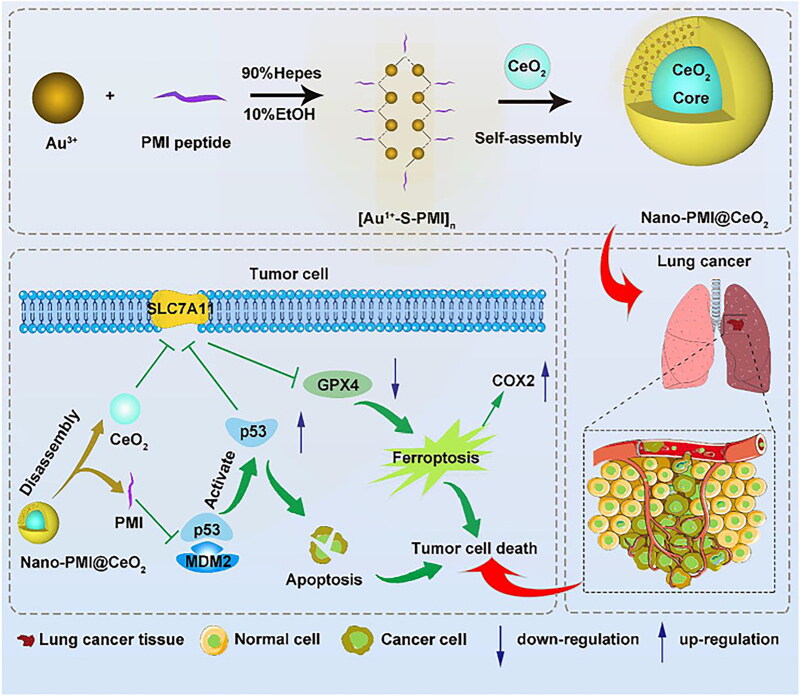
Synthesis Of nanoparticles and mechanism diagram of inducing tumor cell death through P53 signaling pathway. Reproduced with permission. Copyright from wang, et al. 2022, frontiers.

As a tumor suppressor, p53 increases cellular vulnerability to ferroptosis by indirectly influencing iron transport, amino acid metabolism, and antioxidant defense and directly inhibiting SLC7A11 expression [130]. p53 restoration and synergistic ferroptosis were achieved by constructing a metal-organic supramolecular system (Nano-PMI@CeO_2_) with a radical generation module-CeO_2_ NPs as the core and a p53-activating peptide (PMI)-gold precursor polymer as the shell [131]. Nano-PMI@CeO_2_ dramatically decelerated the progression of tumor growth by reactivating the p53 signal pathway and downregulating downstream GPX4 to promote ferroptosis (Figure 8). The experiments showed that the tumour inhibition rate of Nano-PMI@CeO_2_ was greater than 74%, and in survival curve experiments, Nano-PMI@CeO_2_ significantly extended the median survival duration of the mice (26 days), which greatly exceeded the other control groups (19.5 days). This evidence indicated that Nano-PMI@CeO_2_ has a strong anti-cancer effect *in vivo*.

### 6.2. Inhibit gene expression

RNA interference (RNAi) is highly efficient for gene silencing [132,133]. FA/Pt + si-GPX4@IONPs were constructed using a mild triple reaction and coated with Lipofectamine to prevent the degradation of the loaded si-GPX4 [134]. Three primary components realize the routes of attack, namely, Pt, si-GPX4, and IONPs. IONPs were degraded after entering the cells, resulting in enhancement of intracellular Fe^3+^ and Fe^2+^. Pt destroyed mitochondrial DNA and nuclear DNA, leading to the overproduction of H_2_O_2_. Accumulation of intracellular reactants (Fe^2+^, Fe^3+^, and H_2_O_2_) created conditions for efficient ROS production. In addition, co-loaded si-GPX4 knocked out GPX4, which was a key negative regulator of ferroptosis. These factors worked together to promote ferroptosis. Similarly, FesiRNAP NPs consisting of polydopamine as the cloak and Fe^2+^/siRNA as the core were dissociated in response to TME, allowing release of siRNA and tumor-specific Fe^2+^ [135]. Excessive Fe^2+^ in tumor cells triggered ferroptosis, and released siRNA downregulated expression of glyceraldehyde-3-phosphate dehydrogenase to restrain glycolytic pathway and increase Fe^2+^-mediated ferroptotic cell death.

Yes-associated protein (YAP) has been identified as a crucial element of cancer and is a widely known oncogene [136]. YAP signal is connected to the vulnerability of metastasis-prone epithelial cells to ferroptosis *via* Merlin-HIPPO-YAP signal, suggesting a possible connection between YAP and ferroptosis [137]. Zinc oxide NPs (ZONs) exhibited various regulatory functions, such as anti-inflammatory, antibacterial, antidiabetic, anticancer, antiaging, and wound healing [138]. ZONs inhibited the expression of GPX4 and SLC7A11 and elevated the levels of Fe^2+^ and ROS in renal cell carcinoma (RCC) cells, which promoted the occurrence of ferroptosis in tumorigenic mouse models. ZONs also induced miR-27a-3p and downregulated YAP, which promoted ferroptosis [139].

## 7. Energy conversion therapy

### 7.1. Light energy conversion therapy

#### 7.1.1. Photodynamic therapy (PDT) of NPs

PDT is a spatially and temporally regulated anticancer strategy that has been identified as promising. It generates ROS to erode the structure of DNA, proteins, and biomembranes. PDT promotes ROS generation and accumulation. Additionally, the Fenton reaction generates a significant quantity of oxygen, which can reduce hypoxia in tumor tissues and strengthen the effect of PDT [140,141]. Thus, combining PDT with ferroptosis treatment to enhance the anticancer effects is attractive.

PEG-Fns showed light-triggered Fe^2+^ production at the tumor location [142]. Under ordinary blue light, a significant quantity of Fe^2+^ was liberated from PEG-Fns, encouraging iron/ROS-associated GPX4 inhibition and irreversible DNA breakage to promote ferroptosis. These findings highlighted a unique light-controlled Fe^2+^ generation strategy that may completely exploit the anti-cancer potential of Fe^2+^ in chemodynamic and PDT.

Researchers developed NPs that can combine with hypoxic-sensing open probes. This allowed for the activation of hypoxic lighting when needed, leading to a closed-loop process that effectively eliminated the tumour. The assembly of SCNPs was accomplished utilising Srf and a cyanine probe (CNO) that was activated by hypoxia. SRF can be selectively deployed to induce apoptosis in tumour cells located within the normoxic zone. Remarkably, tumour cells in a state of oxygen deprivation were effectively highlighted when nitroreductase lowered CNO in the presence of NADPH. Furthermore, swift exhaustion of NADPH by CNO at the start of fluorescent irradiation hindered the enzymatic conversion of oxidised glutathione (GSSG) to reduce GSH catalysed by GR. The combination of CNO and Srf acted in synergy to inhibit the GSH-GPX4 axis in hypoxic tumour cells, leading to the efficient induction of tumour ferroptosis [143].

#### 7.1.2. Photothermal therapy (PTT) of NPs

PTT produces local heat and noninvasively eliminates tumors [144]. In recent years, a moderate variant of PTT performed at low temperatures has been preferred. A modest PTT may make tumor cell membranes more permeable, which would improve the uptake of Fe^2+^/Fe^3+^ into tumor cells and enhance ferroptosis. The increased local temperature brought on by photothermal conversion also accelerates the Fenton reaction, enhancing ferroptosis [145–150].

A newly developed TSST is an NIR-II molecule with the potential to emit light by aggregation-induced emission (AIE), which was combined with ferrocene and co-assembled docosahexaenoic acid-PEG (DHA-PEG) to form DFT-NPs [151]. DFT-NPs demonstrated heightened capabilities in NIR-II fluorescence, photothermal, ferroptosis, et al. The PTT effect increased Fenton reaction between H_2_O_2_ and ferrocene after endocytosis of NPs, speeding up disintegration of NPs, and triggering the accumulateion of DHA. To investigate the potential anti-cancer properties of DFT-NPs when exposed to light, study conducted *in vitro* tests using the HepG2 cell line. The findings revealed that the apoptosis rate of DFT-NPs reached an impressive 99.8% when exposed to light. The PDX-HCC model was created by injecting tumour blocks from patients into Balb/c nude mice for investigations *in vivo*. DFT-NPs group exhibited a survival rate of 75% following 30 days of exposure to light. A self-synergistic nanomedical medication for image-guided PTT was created through molecular nanoassembly (NA) using DiR (a photothermal probe) and ferrocene (a Fenton reactant Fc). Upon exposure to an laser, the nanoassembly containing DiR caused targeted PTT at specified sites. Furthermore, it greatly enhanced the rate of the Fenton reaction in which fc was involved, and actively encouraged the occurrence of LPO storms. Furthermore, it was worth noting that a substantial quantity of LPOs greatly enhanced sensitivity of cancer cells to PTT by suppressing the increase in cellular HSP90, which triggered ferroptosis. [152].

**Figure 9.**
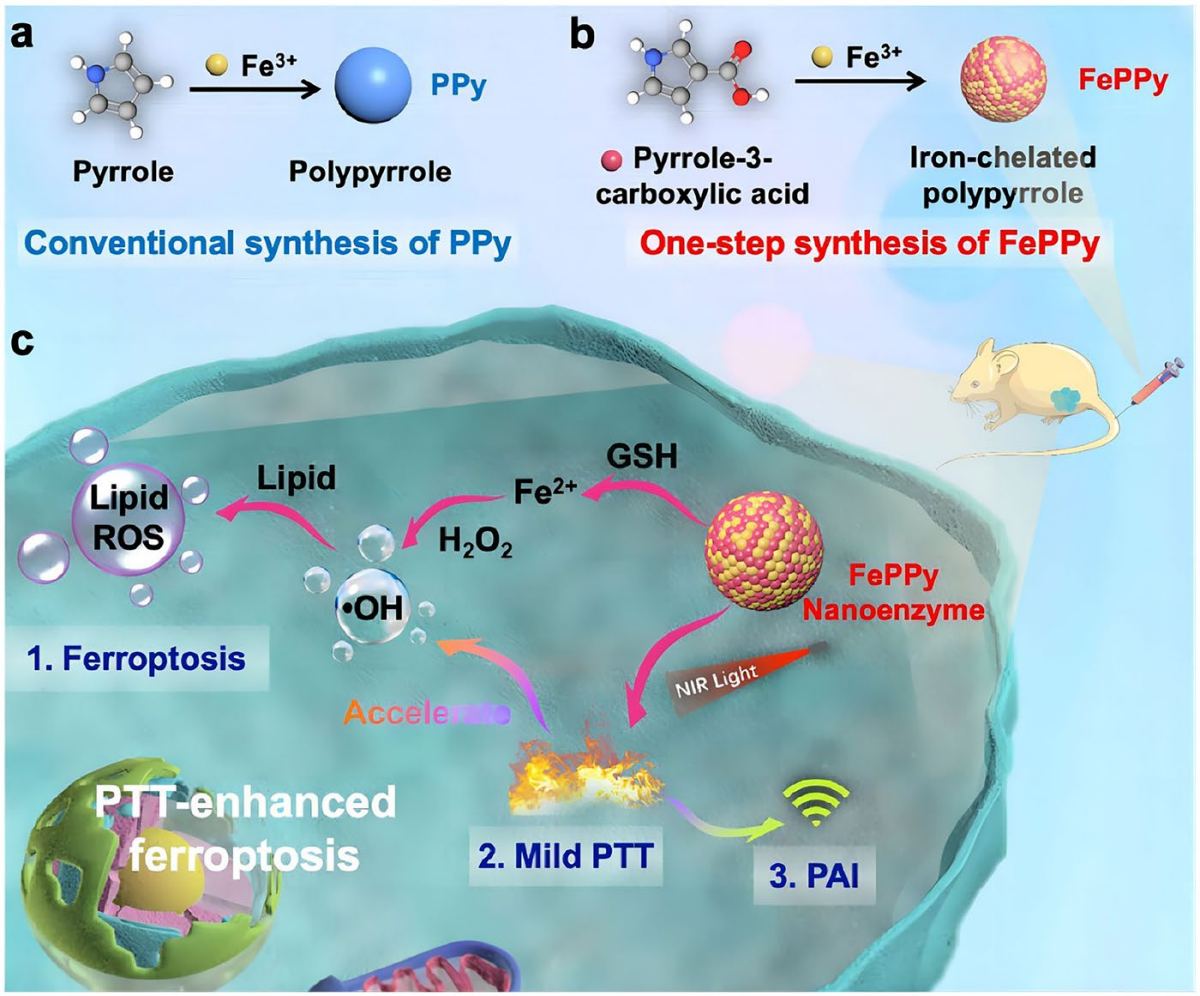
Synthesis Of FePPy and ferroptosis mechanism of FePPy photoinduction. (A,B) Schematic diagram of the synthesis of PPy and FePPy. (C) The mechanism of NPs inducing ferroptosis, including chemical kinetics and photothermal transformation. Reproduced with permission. Copyright 2021, American chemical society.

FePPy NPs were made from iron and pyrrole-3-­carboxylic acid, which worked as an oxidizing agent and monomer [153]. The anticancer effects of FePPy NPs were mainly associated in two ways: first, FePPy NPs decomposed H_2_O_2_ to produce ·OH; second, the NPs activated photothermal transmission utilizing low-temperature PTT, when exposed to laser light, which efficiently accelerated Fenton reactions and boosted ferroptosis, demonstrating the great efficacy of PTT employing these NPs (Figure 9). CuCP Lipo NPs were developed by encapsulating CuCP molecules in a liposome-based nanosystem [154]. The nanosystem displayed inherent nanoenzyme activity and photothermal features owing to the distinctive structure of CuCP Lipo NPs and properties of Cu atoms. CuCP Lipo NPs induced ferroptosis by generating a significant quantity of ·OH *via* Fenton-like reactions, bringing about accumulation of LPO and depletion of GSH. When combined with NIR irradiation, CuCP Lipo NPs enhanced ·OH production, LPO accumulation, and GSH depletion through the photothermal effect. *In vitro* investigations evaluated cell viability in cancer cell lines (MKN45 and CT26) following various treatments. These results indicated that combination of CuCP Lipo NPs and NIR induced the highest level of cell death in two cell lines, with percentages of 77.29% and 75.25% respectively. The results demonstrated that the nanosystem had favourable photothermal characteristics when exposed to laser light, hence successfully enhancing ferroptosis and markedly suppressing tumour growth.

### 7.2. Ultrasonic energy conversion

Owing to its spatiotemporal maneuverability and deep tissue penetration, US-mediated cancer treatment is a potential strategy for treating deep tumors. US is used to induce a cavitation effect, hyperthermia, or ROS to destroy tumor cells [155–157]. The effect of this treatment is still limited, despite its feasibility in various tumor models [158]. Fortunately, nanotechnology has provided effective methods for enhancing the effectiveness of US-mediated cancer therapy. These strategies include improving ultrasonic cavitation, enhancing sonosensitizer delivery, and adjusting the hypoxic TME [159,160].

**Figure 10.**
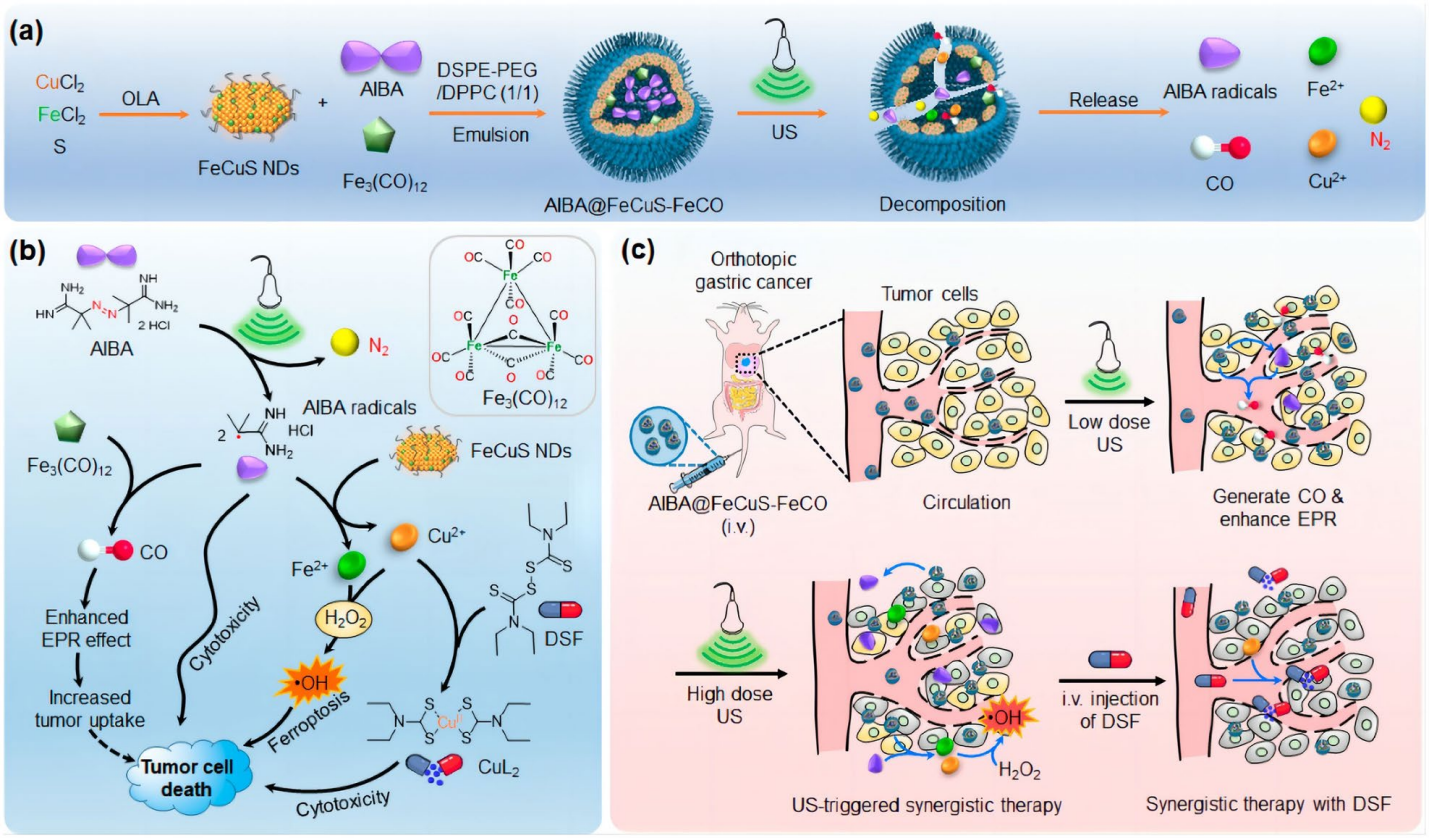
Preparation of AIBA@FeCuS-FeCO and the mechanism of cell death induced by ultrasound. (A,B) The preparation of AIBA@FeCuS-FeCO and synergistic induction of tumor cell death with DSF using US as the medium. (C) Schematic diagram of synergistic treatment of *in situ* gastric neoplasms using US as medium AIBA@FeCuS-FeCO and DSF. Reproduced with permission. Copyright 2021, American chemical society.

AIBA@FeCuS-FeCO, a degradable FeCuS lipid mixture NPs, has a promising future for application in the treatment of deep-seated tumors using synergistic US [161]. NPs were prepared using an amphiphilic phospholipid-assisted emulsion of Fe_3_(CO)_12_, AIBA, and FeCuS NDs. When AIBA@FeCuS-FeCO was exposed to US, higher temperature caused AIBA to decompose into cytotoxic AIBA free radicals and N_2_, which in turn caused NPs to degrade and release Fe^2+^ and Cu^2+^, promoting the Fenton reaction. Furthermore, AIBA radicals directly harmed the cells by reacting with Fe_3_(CO)_12_ to release CO. US showed promise for the treatment of deep cancers because of its accurate temporal-spatial controllability, noninvasiveness, and excellent tissue penetration ability (Figure 10).

This review highlights the current major development in therapeutic types of NPs for inducing ferroptosis in cancer and makes readers clearer about ferroptosis induced by a wide range of NPs for better clinical application (Table 1). Although NPs for ferroptosis induction have made great progress, multiple factors keep on impeding their clinical transformation. For instance, ferroptosis plays a role in intricate metabolic process, encompassing energy and amino acid metabolism, the precise mechanism of which remain incompletely comprehended [9]. Therefore, there is a considerable distance to go to exploit satisfactory NPs for ferroptosis.

**Table 1.** Therapeutic types of functionalized nanoparticles in inducing ferroptosis in cancer therapy.

Therapeutic species	Subtype			Nanoparticle
Classification of nanoparticle properties	Iron oxide NPs			SPIONs [27], IONP-GA/PAA [28], ferumoxytol [31]
	Iron-based conversion NPs			Nanolongan [33]
	Core-shell structure			Fe_3_O_4_@PGL [35], Fe_3_O_4_-SAS@PLT [36], PM-CS [40]
	Organic framework	MOFs	MIL-101(Fe)	MIL-101(Fe)@sor [45], MIL-101(Fe)@RSL3 [46]
			Zifs	DHA/SNP/ICG@Zif-8-FA [54], DOX&GGF@ZIF-8@HA [55]
		COFs		RSL3@COF-Fc [63]
	Silica NPs			Ultrasmall silica NPs [64], Silica NPs [65]
	Lipoprotein NPs			Flip-NPs [66], LDL-DHA NPs [67]
Immunotherapy	Macrophage polarization			ZVI NPs [72], Fe_3_O_4_-SAS@PLT [36],
	Increased T cell infiltration			ZnP@DHA/Pyro-Fe [78]
Loading drugs	Chemical drugs	Chemotherapeutic drug	Dox	PCFD [83]
			Pt	Pt-FMO [91]
			SRF	CuO_2_-PVP-SRF [94], MMSNs@SO [95], SRF@FeShik-GOx-cRGD [96], MNP@PMPC-SRF [97]
			Salinase	HSB-1216 [98]
		Antibiotic		Fe(III)PP@SAS [101]
		Autophagy inducer		TreMMM [104], REFSM [105]
		GPX4 inhibitor		FAS NPs [106]
	Light therapy drugs			Ce6-PEG-HKN_15_ [108], Fe_3_O_4_-PLGA-Ce6 [109], PpIX-PsilQ [112]
	Acoustic therapy drugs			HCIr [113]
Targeted therapy	Biomembrane modification			CM-Fe-siR [117], CM-DSe- SRF-Fe^2+^ [118]
	FA			E/M@FA-LPs [119]
	pH response			TA-Fe/ART@ZIF [120]
	Magnetic targeting			VIO [122]
Multidrug resistance therapy	Regulation of P-gp expression			DFHHP [125]
	Reduce drug efflux			DDTF [126]
	Drug-resistant inhibitors			BSO/CXB@BNP [129]
Gene therapy	Promote gene expression			Nano-PMI@CeO2 [131]
	Inhibit gene expression			FA/Pt + si-GPX4@IONPs [134], FesiRNAP [135], ZONs [139]
Energy conversion therapy	Light energy conversion	Photodynamic therapy		PEG-Fns [142], SCNPs [143]
		Photothermal therapy		DFT-NPs [151], FePPy [153], CuCP Lipo NPs [154]
	Ultrasonic energy conversion			AIBA@FeCuS-FeCO [161]

## Data Availability

Data sharing is not applicable to this article, as no new data were created or analyzed in this study.
